# Nitric Oxide Signaling in Photosynthetic Resilience: Linking Photoprotection and Carbon Gain in Tropical Crops

**DOI:** 10.3390/ijms27198591

**Published:** 2026-09-25

**Authors:** Mohammad Shafiqul Islam, Nusrat Jahan Methela, Young B. Cho, Moon-Sub Lee, Bong-Gyu Mun

**Affiliations:** 1 Department of Agriculture, Noakhali Science and Technology University, Noakhali 3814, Bangladesh; shafik.ag@nstu.edu.bd (M.S.I.); methela.ag@nstu.edu.bd (N.J.M.); 2 Department of Molecular Biosciences & BioEngineering, University of Hawaii at Manoa, Honolulu, HI 96822, USA; ybcho@hawaii.edu; 3 Department of Crop Science, Chungbuk National University, Cheongju 28644, Republic of Korea; 4 Department of Environmental and Biological Chemistry, Chungbuk National University, Cheongju 28644, Republic of Korea

**Keywords:** nitric oxide, S-nitrosylation, chloroplast redox homeostasis, photosynthesis, tropical crops, photoprotection, combined stress, carbon assimilation

## Abstract

Tropical crops are increasingly exposed to climate-driven, recurrent combinations of heat, excessive irradiance, high vapor pressure deficit (VPD), drought, flooding, salinity, and pathogen pressure that collectively impair photosynthesis and reduce crop productivity. Unlike individual stress events, these interacting stresses simultaneously disrupt photoprotection, electron transport, carbon assimilation, stomatal regulation, and chloroplast redox homeostasis, ultimately limiting whole-plant carbon gain under fluctuating environments. Nitric oxide (NO) has emerged as a central signaling molecule coordinating these adaptive responses, yet its roles in photosynthetic regulation remain dispersed across molecular, physiological, and crop-specific studies. This review presents an integrated framework linking chloroplast-level NO signaling with whole-plant photosynthetic performance in major tropical crops, including maize, sugarcane, banana, cassava, and oil palm. We propose that NO regulates photosynthetic resilience through three interconnected axes: (i) reversible protein S-nitrosylation that modulates photosynthetic complexes and Calvin–Benson cycle enzymes; (ii) maintenance of chloroplast redox homeostasis through coordinated reactive oxygen species (ROS) and reactive nitrogen species (RNS) signaling, antioxidant networks, and S-nitrosothiol metabolism; and (iii) ABA-mediated stomatal regulation that optimizes the trade-off between carbon assimilation, stomatal conductance, and water-use efficiency under environmental stress. We further discuss the emerging roles of S-nitrosothiols (SNOs) and S-nitrosoglutathione (GSNO) as central regulators of NO homeostasis and potential biochemical indicators linking chloroplast signaling with whole-plant carbon performance. By synthesizing current evidence across climate-relevant stress combinations, including heat + high light, drought + high VPD, flooding + hypoxia, salinity + pathogen interactions, this review provides a conceptual framework for understanding NO-mediated photosynthetic resilience and identifies key research priorities for developing NO-based strategies to enhance productivity and climate resilience in tropical cropping systems.

## 1. Introduction

Climate variability is placing increasing pressure on agricultural productivity in tropical regions, where crops are routinely exposed to overlapping stresses such as heat, intense light, drought, flooding, salinity, and pathogen attack rather than isolated stress events [1,2,3,4,5]. These simultaneous stresses impose far greater physiological constraints than individual stress factors because they disrupt multiple components of the photosynthetic process at the same time. Damage to photosystem II (PSII), impairment of electron transport, inhibition of Calvin–Benson cycle activity, stomatal limitations, and excessive accumulation of reactive oxygen species (ROS) collectively reduce carbon assimilation and ultimately compromise crop productivity [6,7,8]. Maintaining photosynthetic efficiency under these dynamic conditions has therefore become a major challenge for sustaining crop productivity and improving climate resilience. Despite the corresponding influence of physiological, biochemical, and molecular processes on plant productivity, photosynthesis is the primary process that converts solar energy into chemical energy, supporting plant growth, development, ecosystem productivity, and global food security [9,10,11]. Chloroplasts are the primary sites of photosynthesis, carrying out both the light and dark reactions. Because of their central role in photosynthesis, they are among the first cellular organelles to be affected by abiotic stresses such as heavy metals, nutrient imbalance, hypoxia, UV radiation, and variable light conditions [12,13]. Rising global temperatures, together with other abiotic stresses, pose significant challenges to plant survival by disrupting cellular homeostasis and metabolic processes, ultimately reducing plant growth, biomass accumulation, and crop yield. In tropical agroecosystems, sustaining photosynthetic efficiency has become increasingly difficult because plants are frequently subjected to multiple concurrent environmental stresses, including heat, drought, salinity, flooding, high vapor pressure deficit, and excessive light intensity [14,15,16].

Plants cope with these challenges through highly coordinated signaling networks that integrate environmental perception with metabolic adjustment. Among these signaling molecules, nitric oxide (NO) has emerged as a central regulator of stress acclimation because of its ability to coordinate redox homeostasis, antioxidant defense, hormone signaling, and post-translational protein modification [17,18]. Rather than functioning solely as a reactive nitrogen species, NO acts as a dynamic signaling hub that fine-tunes photosynthetic performance across multiple organizational levels. Within chloroplasts, NO regulates photosynthetic proteins through reversible S-nitrosylation, modulates the balance between ROS and reactive nitrogen species (RNS), and protects the photosynthetic apparatus from oxidative damage [19,20,21]. Beyond the chloroplast, NO integrates abscisic acid (ABA)-dependent stomatal signaling with water-use efficiency and carbon assimilation, thereby linking cellular redox regulation to whole-plant carbon gain under changing environmental conditions [22,23,24,25]. Over the past decade, substantial progress has been made in understanding NO-mediated regulation of photosynthesis. However, much of the available evidence remains fragmented across molecular, biochemical, physiological, and crop-specific studies. Furthermore, most investigations have been conducted under controlled conditions using individual stress factors, whereas tropical crops are typically exposed to multiple interacting stresses that trigger complex and dynamic physiological responses. Consequently, an integrated understanding of how NO coordinates chloroplast function, redox signaling, stomatal behavior, and carbon assimilation under realistic stress combinations is still lacking. In this review, we synthesize current knowledge on the role of NO in regulating photosynthesis under the complex environmental conditions characteristic of tropical cropping systems. We propose an integrated framework in which NO enhances photosynthetic resilience through three interconnected regulatory axes: (i) reversible reprogramming of the photosynthetic machinery via protein S-nitrosylation; (ii) maintenance of chloroplast redox homeostasis through coordinated ROS–RNS interactions and antioxidant networks; and (iii) ABA-mediated regulation of stomatal conductance to optimize carbon assimilation and water-use efficiency. We further discuss crop-specific responses, highlight the emerging significance of S-nitrosothiols (SNOs) and S-nitrosoglutathione (GSNO) as indicators of NO homeostasis, and identify key research priorities for translating NO biology into strategies that improve photosynthetic efficiency, climate resilience, and support sustainable productivity and food security in tropical agriculture as summarized in Figure 1.

## 2. Recurrent Stress Combinations in Tropical Environments

Tropical cropping systems are characterized by dynamic and often interacting environmental stresses rather than isolated stress events. High temperatures frequently coincide with intense solar radiation, drought is commonly accompanied by high atmospheric evaporative demand, and flooding or salinity may occur alongside other abiotic or biotic constraints. These stress combinations disrupt photosynthesis through multiple pathways, including damage to the photosynthetic apparatus, redox imbalance, restricted CO_2_ diffusion, and impaired carbon metabolism. Understanding how plants respond to these complex environments provides the foundation for exploring the regulatory role of NO in maintaining photosynthetic performance and plant productivity.

### 2.1. Heat + High Light

Heat and excessive light intensity disrupt photosynthetic activity. High temperature and light often occur simultaneously in tropical environments, placing exceptional pressure on the photosynthetic machinery [3,26]. While excess light overwhelms the capacity for photochemical energy utilization, elevated temperature accelerates photoinhibition, destabilizes thylakoid membranes, and reduces the efficiency of photosystem II repair [5,27]. Together, these stresses increase reactive oxygen species (ROS) production and limit carbon assimilation, making effective photoprotection essential for sustaining photosynthesis. Maintenance of photosynthesis under supra-optimal temperatures and excessive irradiance is supported by enhanced Rubisco activation, regulated photorespiration, and efficient regeneration of ribulose-1,5-bisphosphate (RuBP) [27].

### 2.2. Drought + High Vapor Pressure Deficit

Water scarcity is frequently intensified by high vapor pressure deficit (VPD), which increases transpirational water loss even when soil moisture is limited. It affects both the light-dependent and light-independent reactions of photosynthesis, leading to severe damage to the photosynthetic apparatus, particularly the photosystems [8]. VPD intensifies transpirational water loss, triggering a series of physiological constraints that impair photosynthesis, suppress growth, and increase vulnerability to carbon starvation and hydraulic failure [28,29]. To conserve water, plants reduce stomatal conductance, but this also restricts CO_2_ uptake and lowers photosynthetic carbon assimilation [6,26]. Prolonged stress further disrupts electron transport and cellular redox balance, increasing the risk of oxidative damage and reducing overall plant productivity [8]. The physiological effects of drought and high VPD are closely linked to multilayered molecular regulation. Plant responses to water deficit involve coordinated transcriptional, post-transcriptional, and epigenetic mechanisms that regulate stomatal behavior, water transport, antioxidant defense, osmotic adjustment, and carbon metabolism [30]. These regulatory layers provide an important molecular context for understanding how NO-mediated signaling may interact with drought-induced changes in photosynthesis and stomatal function.

### 2.3. Flooding and Hypoxia

In most cases, submergence not only induces hypoxic or anoxic conditions within plant tissues but also promotes the accumulation of CO_2_, ethylene, and ammonium leading to profound metabolic and physiological alterations [31]. The restricted diffusion of O_2_ compromises aerobic respiration, while impaired CO_2_ diffusion limits carbon fixation, collectively suppressing photosynthetic performance and plant growth [32]. Reduced oxygen availability limits mitochondrial respiration, disturbs cellular redox homeostasis, and affects nutrient uptake, with secondary consequences for photosynthesis [33]. Recovery following flooding often imposes additional oxidative stress, requiring efficient signaling and metabolic adjustment to restore normal physiological function. Flooding further disrupts nutrient homeostasis, particularly potassium (K^+^) uptake and distribution, thereby exacerbating stress-induced damage. Consistent with this, exogenous K^+^ supplementation has been shown to alleviate the adverse effects of complete submergence by enhancing plant survival and maintaining higher chlorophyll levels compared with plants receiving no additional K^+^ [34].

### 2.4. Salinity and Combined Salinity—Heat/Drought Stress

Salinity imposes both osmotic and ionic stress, reducing water uptake while disrupting ion homeostasis and metabolic activity [35]. Under tropical conditions, salinity is frequently accompanied by heat or drought, creating complex stress environments that intensify oxidative damage and impair photosynthetic efficiency [6,8,35,36,37]. Salinity impairs photosynthesis by altering chloroplast ultrastructure, including changes in chloroplast size, abundance, and lamellar organization, while also affecting lipid and starch accumulation and disrupting transmembrane transport [36]. Thus, combined stresses simultaneously damage photoprotection and carbon assimilation, requiring integrated physiological adjustments, including the maintenance of K^+^/Na^+^ homeostasis and the activation of antioxidant defense systems, to preserve cellular integrity and photosynthetic function [26,37,38,39].

### 2.5. Pathogen Pressure Under Abiotic Stress

Abiotic stress such as drought, salinity and heat, often enhances plant susceptibility to pathogens by impairing physiological performance and altering defense signaling [4,40,41]. In tropical cropping systems, biotic stresses caused by fungal pathogens and plant parasitic nematodes further exacerbate yield losses, particularly when they occur alongside unfavorable environmental conditions [42]. At the same time, pathogen infection further compromises photosynthesis through changes in stomatal behavior, chloroplast function, and resource allocation [40,43]. Plant responses to pathogen infection and abiotic stress share interconnected signaling networks, with reactive oxygen species (ROS) and stress hormones serving as key regulatory hubs [40]. Consequently, exposure to abiotic stresses often increases plant susceptibility to pathogens by disrupting physiological homeostasis and compromising immune responses. The simultaneous occurrence of abiotic and biotic stresses therefore generates a highly complex signaling network in which plants must coordinate growth, defense, and photosynthetic carbon assimilation to maintain physiological performance and ensure survival.

Collectively, these recurrent stress combinations converge on a common outcome: disruption of chloroplast function, redox homeostasis, and carbon assimilation. The following sections examine how NO acts as an integrative signaling molecule to preserve photosynthetic efficiency and coordinate adaptive responses across these interconnected processes.

## 3. Overview of Nitric Oxide Biology in Plants

Nitric oxide (NO) is a highly reactive redox signaling molecule that regulates plant development and adaptation to environmental stress [44,45]. Its biological activity arises largely from interactions with reactive oxygen species (ROS), protein thiols, antioxidants, and hormonal signaling networks, enabling rapid modulation of enzyme activity, redox homeostasis, and cellular responses [17,45,46]. In photosynthetic tissues, NO is increasingly recognized as an important regulator of chloroplast function, where it influences electron transport, photoprotection, antioxidant capacity, and carbon assimilation under stress [25]. Because free NO is short-lived, its biological effects depend on tightly regulated production, compartmentalization, conversion into more stable derivatives, and turnover [47]. These features allow NO signaling to operate across cellular compartments and connect chloroplast responses with broader physiological processes. Importantly, the physiological effects of NO are not uniformly positive and depend strongly on the experimental context. Studies using exogenous NO donors have reported improvements in chlorophyll retention, photosynthetic electron transport, stomatal regulation, and carbon assimilation under different stress conditions; however, the magnitude and direction of these responses vary with donor type, concentration, application route, exposure duration, stress severity, and plant developmental stage. At elevated levels, NO may promote protein tyrosine nitration, disrupt cellular redox balance, and interfere with photosynthetic metabolism rather than providing protection. Moreover, differences in NO detection methods and the transient nature of NO signaling complicate direct comparisons among studies. These variations suggest that NO functions within a concentration- and context-dependent signaling window, in which its effects are determined by the balance between NO, ROS, antioxidant capacity, and downstream signaling pathways. Therefore, evidence for NO-mediated photoprotection should be interpreted in relation to experimental conditions rather than generalized across plant species or stress environments.

### 3.1. NO Production Pathways and Cellular Distribution

Plant NO originates from multiple enzymatic and nonenzymatic pathways whose relative contributions vary with tissue type, developmental stage, and environmental conditions [18,44]. Nitrate reductase-mediated reduction in nitrite represents an important enzymatic source, while additional nitrite-dependent and nonenzymatic pathways can contribute to NO formation, particularly under stress and altered oxygen availability. NO has been detected in chloroplasts, mitochondria, peroxisomes, cytosol, nucleus, and apoplast, reflecting its diverse roles in metabolic and stress signaling [48,49,50]. For the photosynthetic response, its presence within and around chloroplasts is particularly relevant because NO can influence electron transport and redox balance while also interacting with signals originating from other cellular compartments. Thus, the biological significance of NO lies not only in where it is produced but also in how its signal is transmitted and integrated across cellular and whole-plant levels [17]. This integrative function is closely connected with transcriptional reprogramming, as stress-responsive transcription factors coordinate overlapping biotic and abiotic stress pathways and contribute to plant resilience [51]. NO may interact with these regulatory networks, providing an additional signaling layer that links cellular redox changes with broader stress adaptation.

### 3.2. GSNO and SNO Pools as Mobile NO Reservoirs

Because NO is highly reactive and short-lived, plants rely on more stable NO-derived compounds to store, transport, and regulate its signaling activity. Among these compounds, S-nitrosoglutathione and other S-nitrosothiols serve as important reservoirs of NO bioactivity and play a central role in maintaining cellular NO homeostasis [52]. GSNO is formed through the interaction of NO-related species with glutathione and functions as a mobile carrier that extends the signaling capacity of NO beyond its site of production [45,53]. In addition to serving as a storage form, GSNO participates in redox signaling by facilitating protein S-nitrosylation, a reversible post-translational modification that influences the activity, stability, and interactions of numerous proteins [54]. The broader SNO pool similarly acts as a dynamic source of nitrosative signals, allowing plants to rapidly adjust cellular functions in response to developmental and environmental cues [55,56]. The abundance of GSNO and related SNOs is tightly modulated by S-nitrosoglutathione reductase (GSNOR), which controls the balance between NO storage and release [57]. Through its effects on S-nitrosylation status and redox signaling, GSNOR has emerged as a key regulator of plant growth, stress adaptation, and photosynthetic performance [56,57]. Together, GSNO and SNO pools provide an efficient mechanism for stabilizing and distributing NO signals, thereby connecting local cellular responses with broader physiological processes throughout the plant.

## 4. Axis I—Protein S-Nitrosylation Reprograms the Photosynthetic Machinery

Protein S-nitrosylation has emerged as a major mechanism through which NO controls photosynthetic function. This reversible post-translational modification alters the activity, stability, and interactions of target proteins, enabling rapid adjustment of photosynthetic processes in response to environmental fluctuations. The effects of S-nitrosylation extend across multiple components of the photosynthetic mechanism. They include the regulation of PSII repair, electron transport, pigment metabolism, non-photochemical quenching (NPQ), and key enzymes of the Calvin–Benson cycle [25,58,59]. Through these coordinated modifications, NO helps balance energy capture, photoprotection, and carbon assimilation, thereby linking chloroplast redox signaling with whole-plant performance under both optimal and stressful conditions [59]. Light also enhances the GSNOR-mediated degradation of GSNO, the principal mobile reservoir of NO in plants. By regulating GSNO turnover and maintaining optimal levels of S-nitrosothiols, GSNOR plays a critical role in the light-dependent control of histone acetylation and the expression of photosynthesis-related genes [60]. Although S-nitrosylation has been associated with regulation of several photosynthetic proteins, the functional consequences are not necessarily uniform. Depending on the target protein, modification site, cellular redox environment, and duration of NO exposure, S-nitrosylation may either promote protection or alter protein activity in ways that constrain photosynthetic metabolism.

### 4.1. PSII Core Proteins, LHCs, and Repair Dynamics

S-nitrosylation directly influences the stability and turnover of PSII core proteins, including D1 and D2 [25]. Under high light or other stress conditions, these modifications can affect photodamage, protein degradation, and the efficiency of the PSII repair cycle [61]. Light harvesting complexes (LHCs) are also responsive to nitrosative signaling, which may alter energy transfer. By modulating both PSII repair dynamics and LHC function, NO contributes to the maintenance of photochemical efficiency during stress [25,62]. Evidence suggests that NO contributes to maintaining PSII homeostasis by modulating NPQ and stimulating cyclic electron transport, potentially to mitigate photodamage to PSII [63,64]. In maize and sorghum, exogenous NO has been reported to enhance photosynthetic performance by increasing the proportion of open PSII reaction centres and improving their photochemical efficiency [65].

### 4.2. Electron Transport Chain, Plastoquinone Pool, and Cyclic Electron Flow

NO-mediated S-nitrosylation can reprogram electron transport by targeting components associated with the plastoquinone pool and downstream electron carriers [66]. These changes influence the partitioning of electrons between linear and cyclic pathways. Enhanced cyclic electron flow may help maintain ATP supply while limiting over-reduction of the photosynthetic electron transport chain [25]. Such regulation is particularly important under fluctuating light, heat, and drought stress, where redox imbalance can rapidly develop.

### 4.3. Pigment Metabolism and Non-Photochemical Quenching

Pigment metabolism is another important target of nitrosative regulation. S-nitrosylation affects enzymes involved in chlorophyll turnover and carotenoid metabolism, thereby influencing the composition of the light harvesting apparatus. These changes are closely linked with the activation of NPQ, the major photoprotective mechanism that dissipates excess excitation energy as heat. By coordinating pigment remodeling and NPQ responses, NO helps protect the photosynthetic machinery from oxidative damage [25]. Consistent with this regulatory function, Arabidopsis gsnor mutants exhibit altered photosynthetic characteristics, including increased photosystem II (PSII) quantum efficiency and photochemical quenching, accompanied by reduced NPQ [67].

### 4.4. Calvin–Benson Cycle Enzymes and Carbon Assimilation

In addition to regulating the light-dependent reactions of photosynthesis, NO also influences carbon assimilation by modulating key enzymes of the Calvin–Benson cycle [66]. Several Calvin–Benson cycle enzymes are targets of NO-mediated regulation, including Rubisco, Rubisco activase, glyceraldehyde-3-phosphate dehydrogenase (GAPDH), and fructose-1,6-bisphosphatase, have been identified as targets of NO-facilitated regulation through changes in protein abundance or reversible S-nitrosylation [25,67]. Among these, S-nitrosylation of Rubisco and GAPDH suppresses enzymatic activity, providing a direct mechanism by which NO modulates carbon flux [68,69]. Notably, GAPDH also undergoes H_2_O_2_-dependent redox modification, indicating coordinated regulation by reactive oxygen and nitrogen species [20,70]. At first consideration, inhibition of Calvin–Benson cycle enzymes may appear inconsistent with a proposed photoprotective role for NO. However, transient restriction of carbon fixation under severe stress could reduce metabolic demand for ATP and NADPH and redirect carbon and reducing power toward antioxidant defense and stress acclimation. Whether such inhibition is adaptive or detrimental is likely to depend on the intensity and duration of the stress and the extent of NO accumulation. Conversely, persistent or excessive S-nitrosylation could restrict carbon assimilation and contribute to photosynthetic decline. Therefore, NO-mediated regulation of Calvin–Benson cycle enzymes is better understood as a dynamic adjustment of carbon metabolism rather than an inherently positive effect.

## 5. Axis II—Chloroplast Redox Homeostasis and ROS–RNS Balance

Chloroplasts are not only the primary sites of photosynthetic energy conversion but also major sources of reactive oxygen species (ROS). Under optimal conditions, ROS act as essential signaling molecules; however, excessive ROS accumulation disrupts photosynthesis and causes oxidative damage. NO helps maintain chloroplast redox homeostasis by interacting with ROS and modulating reactive nitrogen species (RNS)-dependent signaling. Through reversible redox modifications, antioxidant regulation, and metabolic adjustment, the coordinated ROS–RNS network balances oxidative stress with cellular adaptation, thereby preserving photosynthetic function under adverse environmental conditions [66]. Proteins such as glyceraldehyde-3-phosphate dehydrogenase (GAPDH), which undergo both NO intervened S-nitrosylation and H_2_O_2_-dependent oxidation, exemplify this redox crosstalk and highlight how ROS and RNS coordinately regulate chloroplast metabolism during stress [20,70]. NO does not simply act as an antioxidant signal. Its interaction with ROS can either facilitate redox acclimation or promote oxidative/nitrosative damage when ROS–RNS production exceeds the capacity of antioxidant systems.

### 5.1. ROS Sources in Chloroplast and NO Interactions

Photosynthetic electron transport inevitably generates reactive oxygen species (ROS), particularly when absorbed light energy exceeds the capacity for carbon assimilation. Consequently, chloroplasts produce superoxide, hydrogen peroxide, and singlet oxygen through photosystem I (PSI), photosystem II (PSII), and associated electron transport components, with ROS accumulation increasing under high light, heat, drought, salinity, and pathogen stress [3,40,71,72]. Although excessive ROS can impair photosynthesis and cause oxidative damage, they also function as essential signaling molecules that coordinate stress adaptation [72]. NO fine-tunes this redox balance by modulating ROS production through direct chemical reactions and the regulation of electron transport and antioxidant defenses [73]. During pathogen infection, chloroplasts are among the earliest sites of NO accumulation, contributing to immune signaling and the activation of pattern-triggered immunity (PTI), effector-triggered immunity (ETI), hypersensitive response-associated programmed cell death, and defense gene expression [71,73,74,75]. Crosstalk between NO and ROS further generates reactive nitrogen species, such as peroxynitrite (ONOO^−^), while competing for common molecular targets, including protein thiols, thereby shaping stress-specific redox signaling [21,76,77,78]. In parallel with salicylic acid (SA), ROS and NO also contribute to the establishment of systemic acquired resistance (SAR) [79]. Consistent with these roles, proteomic studies in citrus have identified photosynthetic proteins, particularly Calvin–Benson cycle enzymes, as major targets of coordinated H_2_O_2_ and NO-dependent regulation under nitro-oxidative stress, highlighting the central role of chloroplast redox signaling in integrating photosynthesis with plant immunity and environmental stress adaptation [67].

### 5.2. Peroxynitrite and Tyrosine Nitration Versus S-Nitrosylation

The reaction of NO with superoxide generates peroxynitrite, a highly reactive nitrogen species capable of modifying proteins, lipids, and nucleic acids [66,78]. One major consequence of ONOO^−^ formation is protein tyrosine nitration, which is generally associated with irreversible or persistent alterations in protein function under severe oxidative stress. In contrast, S-nitrosylation is a reversible redox-dependent modification in which an NO group is covalently attached to reactive cysteine residues, enabling dynamic regulation of protein activity, stability, localization, and protein–protein interactions [67]. The balance between tyrosine nitration and S-nitrosylation therefore reflects the cellular redox environment and often determines whether NO promotes stress acclimation or contributes to oxidative injury. S-nitrosylation is tightly regulated by GSNOR, which maintains intracellular S-nitrosothiol homeostasis [80]. Recent advances in plant S-nitrosoproteomics have revealed that numerous proteins involved in photosynthesis, antioxidant defense, hormone signaling, and stress responses are subject to this reversible modification, enabling rapid metabolic adjustment under changing environmental conditions [54,67,81]. For the period of pathogen infection, NO accumulates rapidly and S-nitrosylates several chloroplast proteins, including the PSII oxygen-evolving complex and the Rubisco large subunit, linking effector-triggered immunity (ETI) with photosynthetic regulation [70,82,83]. In short, reversible S-nitrosylation highlights as a key mechanism through which NO integrates chloroplast redox homeostasis, photosynthetic function, and stress adaptation.

### 5.3. Antioxidant Networks (AsA–GSH, SOD, CAT, APX, Trx/Prx)

The antioxidant network constitutes the primary defense against excessive reactive oxygen species (ROS) and functions in close coordination with NO to maintain chloroplast redox homeostasis [84,85]. The ascorbate–glutathione (AsA–GSH) cycle, together with superoxide dismutase (SOD), catalase (CAT), ascorbate peroxidase (APX), and the thioredoxin/peroxiredoxin (Trx/Prx) system, forms an integrated antioxidant machinery that scavenges ROS while preserving the redox signaling required for stress acclimation [84,86,87]. Beyond enzymatic detoxification, ascorbate and glutathione also interact with reactive nitrogen species (RNS), including peroxynitrite (ONOO^−^), thereby limiting nitro-oxidative damage and helping to maintain cellular redox balance [66]. Although the physiological significance of some of these reactions remains to be fully resolved, the high intracellular concentrations of these antioxidants are thought to buffer excessive NO-derived species under non-stress conditions. Glutathione plays a particularly important role in NO signaling through the formation of GSNO, the major intracellular reservoir and transport form of NO [25]. GSNO functions as a mobile source of bioactive NO and facilitates protein S-nitrosylation, thereby linking glutathione metabolism with redox signaling [84,88]. Cellular GSNO levels are tightly controlled by S-nitrosoglutathione reductase, which regulates S-nitrosothiol homeostasis and indirectly influences protein denitrosylation [45,87]. Through these mechanisms, NO coordinates the activity of antioxidant enzymes and the AsA–GSH cycle via transcriptional regulation and reversible post-translational modifications, strengthening chloroplast antioxidant capacity, minimizing oxidative damage, and sustaining photosynthetic performance under environmental stress [25,84]. The major components of NO-associated enzymes are summarized in Table 1.

## 6. Axis III—ABA-Linked Stomatal Regulation and Whole-Plant Carbon Gain

Photosynthetic performance depends not only on the efficiency of chloroplast metabolism but also on the regulation of gas exchange at the leaf surface. Stomata continuously balance two competing demands: allowing sufficient CO_2_ to enter for carbon assimilation while minimizing water loss through transpiration. NO plays a pivotal role in this process by acting downstream of abscisic acid (ABA) and integrating hormonal, redox, and calcium signaling. Through this coordinated regulation, NO enables plants to optimize carbon gain under favorable conditions while improving water conservation and stress resilience when environmental conditions become limiting. NO-mediated stomatal closure can improve water conservation under drought, but sustained closure may simultaneously restrict CO_2_ diffusion and reduce carbon assimilation. Therefore, the physiological outcome depends on the balance between water conservation and maintenance of photosynthetic carbon gain.

### 6.1. NO–ABA–Ca^2+^ Signaling in Guard Cells

The regulation of photosynthetic performance involves coordinated control of chloroplast metabolism and stomatal aperture, which determines the balance between CO_2_ uptake for carbon assimilation and water loss through transpiration. NO is a focal component of this process, acting downstream of abscisic acid (ABA) to integrate hormonal, redox, and Ca^2+^ signaling [89]. Through this coordinated network, NO optimizes water-use efficiency while sustaining photosynthetic carbon gain under fluctuating environmental conditions. ABA-induced stomatal closure is initiated by the activation of plasma membrane NADPH oxidases (RBOHD/F), leading to the rapid production of hydrogen peroxide (H_2_O_2_), which acts as an early second messenger in guard cells [90,91,92]. H_2_O_2_ is perceived by the extracellular receptor kinase HPCA1, triggering Ca^2+^ influx and downstream signaling that promotes NO production, primarily through nitrate reductase (NR) [23]. Nitric oxide subsequently amplifies guard cell signaling by regulating Ca^2+^ release, cyclic GMP (cGMP)-dependent pathways, and ion channels, including outward rectifying K^+^ channels through S-nitrosylation, ultimately promoting guard cell turgor loss and stomatal closure [22,23]. Genetic evidence from Arabidopsis further provides strong evidence for an association of mutations in RBOHD/F, HPCA1, or NR (nia1/nia2) impair ABA-induced ROS production, NO accumulation, Ca^2+^ signaling, and stomatal closure, confirming the tight functional integration of these signaling components [23,90,91,93]. The protein phosphatases ABI1 and ABI2 also function downstream of NO to fine-tune ABA signaling in guard cells [22]. Beyond serving as a downstream signal, NO also functions as a key regulator of cytosolic Ca^2+^ dynamics, thereby linking ABA perception with the activation of ion transport required for stomatal closure [89]. Through interactions with Ca^2+^ channels, cyclic GMP (cGMP), and cyclic ADP ribose (cADPR)-dependent signaling, NO promotes transient increases in cytosolic Ca^2+^ concentration ([Ca^2+^]_(_cyt_)_), which act as central integrators of guard cell responses [94].

Rather than functioning as a simple on–off signal, changes in [Ca^2+^]_(_cyt_)_ generate distinct spatiotemporal Ca^2+^ signatures characterized by differences in amplitude, frequency, duration, and spatial distribution [91,92]. These signatures arise through the coordinated activity of plasma membrane Ca^2+^ channels, intracellular Ca^2+^ stores, transporters, and buffering systems, and encode information from ABA, NO, elevated CO_2_, and pathogen-derived signals [95,96,97]. The encoded Ca^2+^ signals are subsequently decoded by Ca^2+^ sensor proteins, including calmodulins (CaMs/CMLs), calcineurin B-like proteins (CBLs), CBL-interacting protein kinases (CIPKs), and calcium-dependent protein kinases (CDPKs), which regulate downstream ion channels and accelerate guard cell turgor loss and stomatal closure [97,98,99]. Experimental studies further demonstrate that both the amplitude and frequency of Ca^2+^ oscillations determine the efficiency and duration of stomatal closure [100]. Only oscillatory patterns within specific quantitative ranges effectively activate anion channels and sustain pore closure, indicating that guard cells function as sophisticated information processing systems rather than passive responders to Ca^2+^ influx. The integration of ABA, ROS, NO, and Ca^2+^ signaling also extends to plant immunity, where pathogen-associated molecular patterns (PAMPs) and microbial elicitors induce rapid ROS and NO accumulation to promote stomatal closure and restrict pathogen entry [92]. The convergence of ABA, ROS, NO, and Ca^2+^ signaling highlights a common regulatory network that integrates abiotic and biotic stress responses [22,23,90,91,92]. By dynamically regulating stomatal conductance in response to environmental signals, NO may contribute to balancing CO_2_ uptake with transpirational water loss, thereby supporting carbon gain while limiting excessive water loss under stress.

### 6.2. Tradeoffs Between Assimilation, Conductance, and Water Use Efficiency

Stomatal regulation certainly involves tradeoffs between carbon gain and water conservation. Partial stomatal closure reduces transpirational water loss and often improves intrinsic water-use efficiency (WUE), but excessive or prolonged closure restricts CO_2_ diffusion and limits photosynthetic carbon assimilation (A). Consequently, the physiological outcome depends on the timing, magnitude, and duration of stomatal responses [101]. Rather than inducing sustained stomatal closure, NO fine-tunes stomatal conductance (g_s_), enabling plants to optimize the balance between CO_2_ uptake, water loss, and photosynthetic performance under fluctuating environmental conditions. Benefits of NO extend well beyond guard cell signaling. Under favorable conditions, exogenous NO donors such as sodium nitroprusside enhance stomatal conductance, thereby increasing CO_2_ diffusion into the leaf and promoting higher net photosynthetic rates without proportionally increasing transpiration [102]. As a result, improvements in carbon assimilation are often accompanied by enhanced intrinsic WUE, indicating that NO optimizes gas exchange rather than simply stimulating stomatal opening [7]. Protective effects of NO involve the maintenance of cellular membrane integrity, stabilization of the photosynthetic mechanism, and preservation of metabolic function under stress. Exogenous NO has been associated with membrane stability during water deficit [103,104] and alleviates heat-induced impairment of PSII photochemistry in rice, although the extent of protection varies with plant species and NO donor concentration [105,106]. In some cases, improved photosynthetic performance occurs even without substantial recovery of PSII activity, implying that the maintenance of extra-thylakoid membrane integrity and cellular metabolism may contribute more to stress tolerance than direct protection of the thylakoid membranes [102]. Nitric oxide has also been linked to the regulation of photorespiration and respiratory metabolism. Under heavy metal stress, such as arsenic and cadmium exposure, NO reduces photorespiration while promoting the alternative respiratory pathway, thereby optimizing energy utilization and mitigating oxidative damage [7,107,108,109].

At the chloroplast level, NO helps sustain carbon fixation by protecting the photosynthetic apparatus and maintaining Calvin–Benson cycle activity during stress. In salt-stressed tomato seedlings, endogenous NO preserved photosynthetic reaction centers, improved photochemical efficiency, and enhanced net photosynthesis (Pn), whereas scavenging NO with hemoglobin (Hb) largely abolished these protective effects and diminished glutathione (GSH) mediated improvements in carbon assimilation. These findings demonstrate that the beneficial effects of GSH on photosynthesis depend largely on NO signaling [110]. Mechanistically, NO supports both CO_2_ carboxylation and ribulose-1,5-bisphosphate (RuBP) regeneration by maintaining the activity and expression of key Calvin–Benson cycle enzymes, including phosphoglycerate kinase (PGK), glyceraldehyde-3-phosphate dehydrogenase (GAPDH), sedoheptulose-1,7-bisphosphatase (SBPase), fructose-1,6-bisphosphatase (FBPase), fructose-bisphosphate aldolase (FBA), and transketolase (TK), thereby sustaining carbon flux and alleviating feedback inhibition of photosynthesis under salinity stress [111,112,113,114,115]. Thus, NO optimizes whole-plant carbon gain through the coordinated regulation of both stomatal conductance and mesophyll metabolism, enabling a dynamic balance between assimilation, g_s_, and WUE across changing environmental conditions. Ultimately, NO integrates stomatal behavior with chloroplast metabolism, providing a mechanistic bridge between cellular redox regulation and whole-plant carbon gain under environmental stress.

The integration of recurrent environmental stresses with NO-mediated S-nitrosylation, ROS–RNS homeostasis, SNO/GSNO metabolism, and ABA-linked stomatal regulation, and their combined contribution to photoprotection, carbon assimilation, water-use efficiency, and stress resilience, is summarized in Figure 2.

## 7. Crop-Specific Integration in Tropical Systems

The core regulatory functions of nitric oxide are broadly conserved across plants, but their physiological outcomes vary among tropical crops owing to differences in photosynthetic pathways, developmental stages, and adaptive responses to environmental stress. These species-specific characteristics influence the production, storage, and signaling of NO, ultimately determining its role in photoprotection, redox regulation, and carbon assimilation. Therefore, translating mechanistic insights into practical strategies requires a crop-specific perspective that recognizes both shared regulatory mechanisms and unique physiological responses. The integration of these NO-mediated regulatory mechanisms across representative tropical crops is summarized in Table 2, highlighting how chloroplast protection, redox regulation, stomatal control, and carbon assimilation contribute to crop-specific stress responses. Comparative analyses across representative tropical crops can reveal conserved patterns of NO action while identifying species-specific traits that may be exploited to improve photosynthetic performance and stress resilience.

As summarized in Table 2, the relative contribution of these NO-mediated processes varies among tropical crops, reflecting differences in stress sensitivity, photosynthetic regulation, and adaptive capacity. The crop-specific responses summarized in Table 2 also indicate that the photosynthetic context of NO signaling differs among tropical crops. In the C_4_ crops maize and sugarcane, NO-mediated responses are associated with processes that are particularly relevant to maintaining photosynthetic performance under high evaporative or thermal demand. In maize, for example, the emphasis is on PSII stability and electron transport under combined heat and high-light stress [116], whereas in sugarcane, NO-mediated regulation is more closely associated with stomatal control and cyclic electron transport under drought and high VPD [117,118]. These differences may reflect the distinct physiological constraints imposed by the respective stress combinations rather than fundamentally different NO signaling pathways.

In C_3_ crops, the apparent targets are broader and include chloroplast function, pigment stability, carbon assimilation, and interactions between photosynthetic and stress-defense processes. Banana is represented by chloroplast function and recovery under flooding or hypoxia [17], while cassava is associated with pigment stability and carbon assimilation under combined salinity and heat [119]. Oil palm represents a different physiological context as a woody perennial, where photosynthetic regulation occurs alongside longer-term investment in structural growth and defense under recurrent environmental stresses [120]. Thus, Table 2 highlights differences in the relative physiological targets of NO across crop types while also showing considerable overlap in core processes such as photoprotection, redox regulation, stomatal control, and carbon assimilation.

### 7.1. Baseline SNO/GSNO Pools Across Organs and Developmental Stages

The regulatory capacity of NO depends not only on its production but also on the abundance and distribution of S-nitrosothiols and S-nitrosoglutathione, which serve as dynamic reservoirs of NO bioactivity. Their levels vary among organs and throughout plant development, reflecting differences in metabolic activity, redox status, and physiological function. Consequently, young and mature leaves, roots, stems, and reproductive tissues each possess distinct NO signaling environments [102,121]. Establishing baseline SNO and GSNO profiles across tissues and developmental stages will provide a valuable reference for distinguishing constitutive NO homeostasis from stress-induced responses, thereby enabling more meaningful comparisons among tropical crop species.

### 7.2. Stress-Specific NO Intervention Points Across Photosynthetic Steps

Environmental stresses such as heat and high light disrupt photosynthesis through distinct physiological and biochemical pathways, and the regulatory role of NO changes accordingly. Excess light primarily threatens photoprotection, drought restricts stomatal conductance and carbon assimilation, whereas heat and salinity impair both electron transport and metabolic processes [7,110]. As a result, the primary targets of NO differ with the nature of the stress, ranging from protein S-nitrosylation and chloroplast redox regulation to antioxidant defense and stomatal signaling. Defining these stress-specific intervention points will help identify the photosynthetic processes that benefit most from NO regulation and provide a mechanistic framework for improving photosynthetic resilience across diverse tropical cropping systems.

### 7.3. Standardized Metrics for Cross-Crop Comparison

Progress in NO research is often constrained by differences in experimental design, measurement techniques, and physiological indicators. As a result, direct comparisons among crop species remain difficult. Standardized metrics that integrate NO-related metabolites, protein modifications, chlorophyll fluorescence, gas exchange, antioxidant capacity, and carbon assimilation would facilitate more robust cross-study and cross-crop analyses. Such an approach would strengthen mechanistic interpretation, improve reproducibility, and accelerate the translation of laboratory findings into field relevant strategies for enhancing productivity in tropical crops. To improve reproducibility and facilitate meaningful comparisons across species and environments, future studies should adopt a common set of physiological, biochemical, and analytical indicators for evaluating NO-mediated regulation of photosynthesis (Table 3).

The proposed framework is not intended to replace crop-specific or stress-specific measurements but to establish a common dataset that improves data comparability across studies. Its adoption would facilitate meta-analyses, strengthen mechanistic synthesis, and accelerate the development of NO-based strategies for improving photosynthetic efficiency and climate resilience in tropical crops. Emerging approaches that precisely manipulate carbon partitioning further highlight the potential to optimize carbon gain and stress resilience while maintaining crop productivity under heat stress [122]. In this context, NO-mediated regulation of photosynthetic carbon assimilation and stomatal behavior may provide an additional signaling layer for balancing carbon gain with water loss under combined environmental stresses.

## 8. Knowledge Gaps and Future Directions

Although considerable progress has been made in understanding NO-mediated regulation of photosynthesis, several important questions remain unresolved. Most current knowledge is based on controlled experiments involving individual stress factors and a limited number of plant species, whereas tropical crops are routinely exposed to multiple, interacting environmental stresses. Future research should therefore move beyond single stress models and adopt integrated approaches that better reflect the complexity of tropical production systems. Such efforts will strengthen mechanistic understanding and facilitate the development of NO-based strategies for improving crop resilience and productivity.

### 8.1. Standardization of SNO Quantification

Reliable quantification of S-nitrosothiols (SNOs) remains challenging because analytical methods differ in sensitivity, specificity, and sample preparation. Established protocols for SNO and GSNO quantification provide useful frameworks for improving analytical consistency [123]. Wider adoption of validated methods, together with standardized sampling, normalization, and reporting practices, would improve reproducibility and enable more meaningful comparisons of SNO dynamics across stress conditions and crop species.

### 8.2. Systematic Mapping of S-Nitrosylated Photosynthetic Proteins

Although numerous photosynthetic proteins have been identified as potential targets of S-nitrosylation, the chloroplast nitrosoproteome remains incompletely characterized. Future studies integrating quantitative proteomics with functional analyses will help identify key regulatory targets, resolve the dynamics of protein modification under different stress conditions, and clarify how S-nitrosylation coordinates photoprotection, electron transport, and carbon assimilation.

### 8.3. Experimental Designs Reflecting Combined Tropical Stress Scenarios

Most studies evaluate heat, drought, salinity, flooding, or pathogen infection independently, despite their frequent co-occurrence in tropical environments. Multifactorial experiments that combine physiological, biochemical, and molecular analyses under realistic stress combinations will provide a more comprehensive understanding of NO signaling and improve the translation of laboratory discoveries into field applications.

## 9. Conclusions

Nitric oxide has emerged as a crucial regulator that links chloroplast function with whole-plant photosynthetic performance under the complex stress combinations characteristic of tropical cropping systems. Rather than acting through a single pathway, NO integrates protein S-nitrosylation, redox homeostasis, antioxidant defense, and ABA-mediated stomatal regulation to protect the photosynthetic machinery, optimize carbon assimilation, and improve water-use efficiency. Together, these interconnected processes enable plants to maintain photosynthetic efficiency under heat, drought, salinity, flooding, excessive light, and pathogen pressure. This review highlights NO as a molecular bridge connecting chloroplast-level regulation with whole-plant carbon gain through three interconnected regulatory axes. At the same time, it emphasizes that our current understanding is still largely based on single stress experiments and a limited number of model species. Future research should focus on combined stress scenarios representative of tropical environments, expand investigations to major tropical crops, and integrate advanced redox proteomics, S-nitrosoproteomics, and physiological phenotyping with standardized measurements of NO, SNOs, GSNO, and photosynthetic traits. A deeper understanding of NO signaling across the chloroplast to whole-plant continuum will not only advance our understanding of plant stress adaptation but also support the development of climate resilient crops with improved photosynthetic efficiency and sustainable productivity under changing environmental conditions.

## Figures and Tables

**Figure 1:**
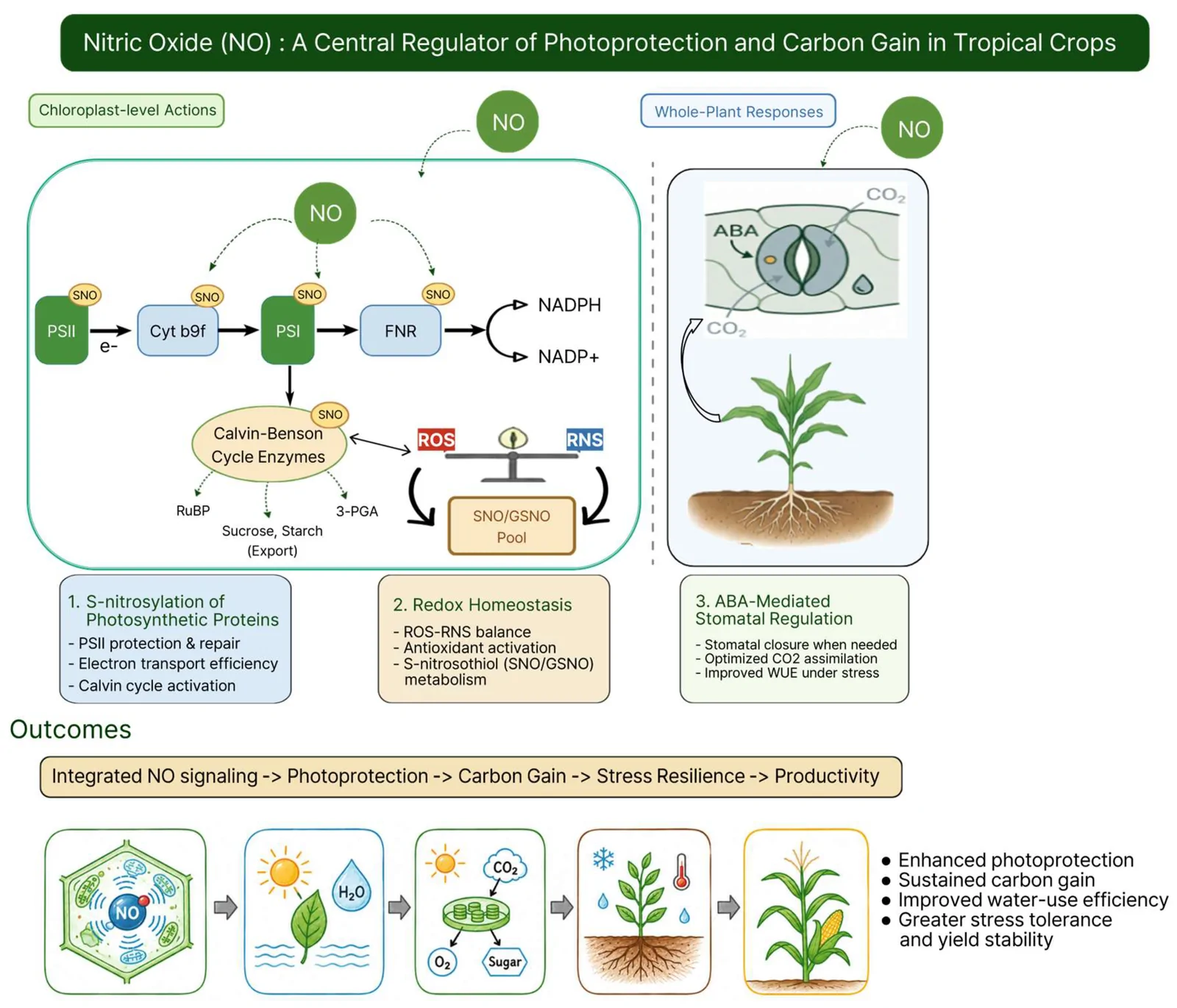
Integrated model of nitric oxide (NO)-mediated regulation of photoprotection and carbon gain in tropical crops. NO coordinates photosynthetic resilience through interconnected regulation of protein S-nitrosylation, chloroplast ROS–RNS/redox homeostasis, and ABA-linked stomatal signaling. Together, these processes connect cellular NO signaling and photoprotection with carbon assimilation, water-use efficiency, stress resilience, and whole-plant performance.

**Figure 2:**
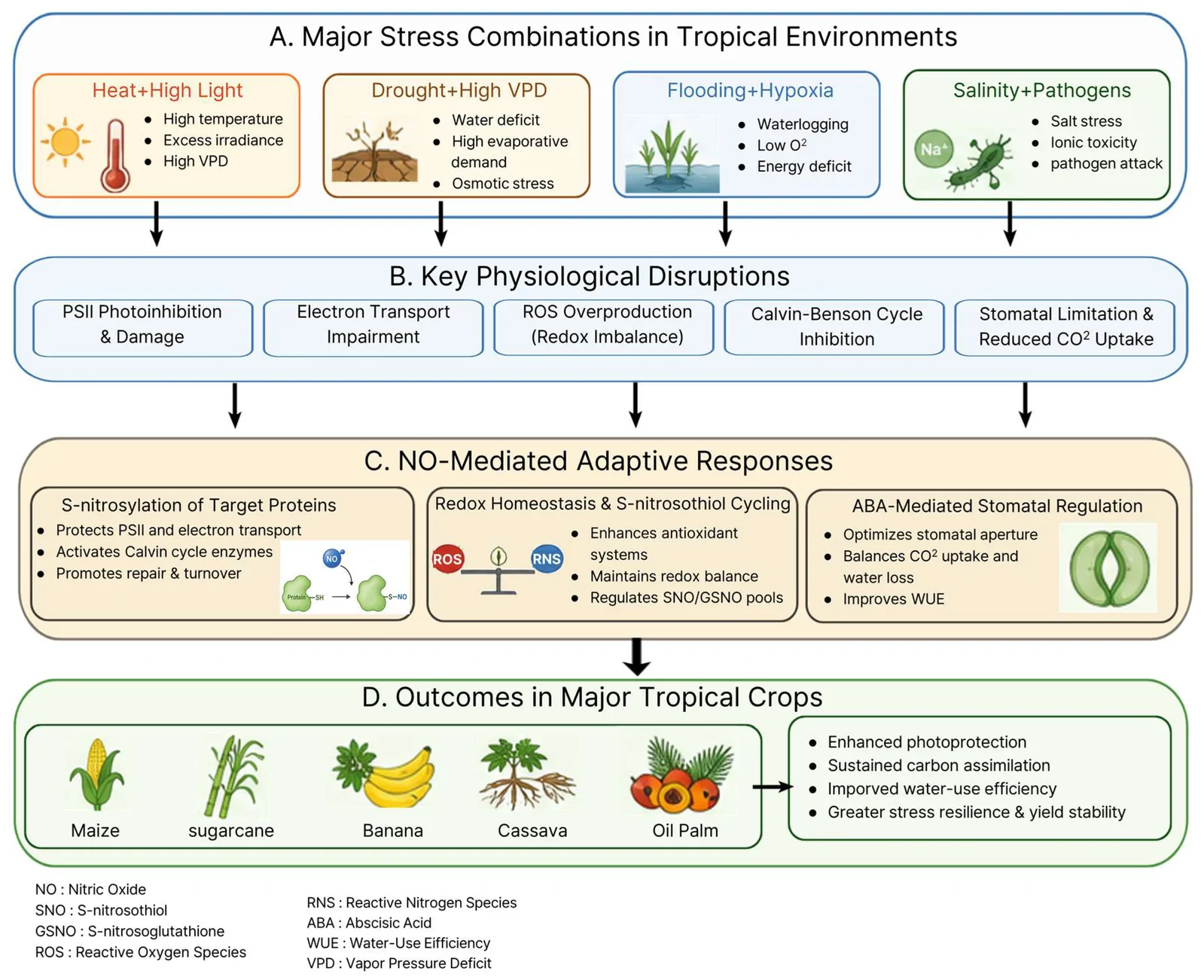
Conceptual framework linking recurrent environmental stress combinations with nitric oxide (NO)-mediated photosynthetic adaptation in major tropical crops. Combined stresses converge on photoinhibition, redox imbalance, impaired electron transport and carbon metabolism, and stomatal limitation. NO-mediated S-nitrosylation, ROS–RNS homeostasis, SNO/GSNO metabolism, and ABA-linked stomatal regulation collectively contribute to photoprotection, carbon assimilation, water-use efficiency, and stress resilience. Abbreviations: VPD, vapor pressure deficit; PSII, photosystem II; ROS, reactive oxygen species; CO_2_, carbon dioxide; NO, nitric oxide; RNS, reactive nitrogen species; SNO, S-nitrosothiols; GSNO, S-nitrosoglutathione; ABA, abscisic acid; WUE, water-use efficiency.

**Table 1:** Major antioxidant enzymes involved in NO-mediated redox regulation under plant stress.

Antioxidant Component	Major Function	Relationship with NO/NO-Derived Signals
SOD	Converts O_2_^−^ to H_2_O_2_	NO can modulate SOD activity/expression, contributing to control of superoxide accumulation
CAT	Decomposes H_2_O_2_ to H_2_O and O_2_	NO can influence CAT activity and expression, supporting H_2_O_2_ homeostasis
APX	Removes H_2_O_2_ using ascorbate	NO-associated signaling can enhance APX activity, strengthening H_2_O_2_ detoxification
GR	Regenerates reduced glutathione (GSH) from GSSG	NO can affect the cellular GSH/GSSG balance and thereby influence glutathione-dependent ROS detoxification
MDHAR/DHAR	Maintains the reduced ascorbate pool	NO–ROS interactions can modify the ascorbate redox cycle and antioxidant capacity
Trx/Prx	Maintains protein redox state and removes peroxides	NO can interact with thiol-based redox systems through S-nitrosylation and related redox modifications
GSNOR	Regulates cellular GSNO/SNO levels	Controls NO bioactivity by regulating GSNO turnover and thereby influences S-nitrosylation-dependent signaling

Abbreviations: APX, ascorbate peroxidase; CAT, catalase; DHAR, dehydroascorbate reductase; GSH, reduced glutathione; GSSG, oxidized glutathione; GSNOR, S-nitrosoglutathione reductase; MDHAR, monodehydroascorbate reductase; NO, nitric oxide; Prx, peroxiredoxin; SNO, S-nitrosothiol; SOD, superoxide dismutase; Trx, thioredoxin.

**Table 2:** Integration of nitric oxide-mediated regulation of photosynthesis across representative tropical crops.

Crop	Photosynthetic Type	Predominant Stress Combination	Primary Stress Factor	Primary Photosynthetic Target	NO Intervention	Representative Physiological Indicators	Evidence for SNO/GSNO
Maize	C_4_	Heat + High Light	Thermal/Light	PSII stability, electron transport (ETR)	NO donor/scavenger	Fv/Fm, ΦPSII, NPQ, A	Increased SNO accumulation
Sugarcane	C_4_	Drought + High VPD	Water deficit	Stomatal regulation, cyclic electron transport (CET)	NO–ABA signaling	A, g_s_, WUE, CET	GSNO modulation
Banana	C_3_	Flooding/Hypoxia	Low O_2_	Chloroplast function and recovery	NO signaling	Chl, A	SNO accumulation
Cassava	C_3_	Salinity + Heat	Ionic/Osmotic	Pigment stability and carbon assimilation	NO donor	Fv/Fm, chlorophyll, Rubisco activity	Limited reports
Oil palm	C_3_	Pathogen + Heat	Biotic	Photosynthesis defense balance	NO modulation	A, defense markers	Remains scarce

This table highlights the current understanding of NO-mediated regulation of photosynthesis across representative tropical crops exposed to recurrent stress combinations. While the fundamental mechanisms of NO signaling appear to be largely conserved, their physiological consequences differ among species because of variations in stress sensitivity, photosynthetic characteristics, and developmental adaptation. The table also reveals important gaps in the current literature, particularly the limited information on SNO/GSNO homeostasis and the uneven distribution of evidence across economically important tropical crops. Addressing these gaps will be essential for developing a unified framework that links NO signaling with crop-specific improvements in photosynthetic efficiency and productivity.

**Table 3:** Proposed framework for standardized assessment of NO-mediated photosynthetic responses in tropical crops.

Module	Core Indicators	Recommended Method/Instrument	Reporting Standard	Essential Experimental Metadata	Quality Considerations
Gas exchange	A, g_s_, Ci	IRGA (LI-6800)	µmol CO_2_ m^−2^ s^−1^	PPFD, leaf temperature, VPD, ambient CO_2_	Standardize chamber conditions and VPD/light
Chl fluorescence	Fv/Fm, ΦPSII, NPQ, ETR	PAM fluorometer	Dimensionless or µmol e^−^ m^−2^ s^−1^	Dark adaptation period, actinic light intensity	Fixed protocol
Redox status	H_2_O_2_, AsA/DHA, GSH/GSSG	Fluorometric or spectrophotometric assays	nmol g^−1^ FW	Sampling time, tissue type, storage conditions	Rapid sample quenching and processing
NO homeostasis	NO, GSNO, total SNO	Chemiluminescence, LC–MS/MS, biotin switch assays	pmol–nmol g^−1^ FW	Tissue pH, light conditions, metal chelators	Avoid artifacts
Productivity	Biomass, WUE, Yield	Growth and yield analysis	g plant^−1^, kg ha^−1^	Plant density, developmental stage, field or pot conditions	controlled environment and field experiments

## Data Availability

No new data were created or analyzed in this study. Data sharing is not applicable to this article.
